# Fiber Optic Sensing Technologies for Battery Management Systems and Energy Storage Applications

**DOI:** 10.3390/s21041397

**Published:** 2021-02-17

**Authors:** Yang D. Su, Yuliya Preger, Hannah Burroughs, Chenhu Sun, Paul R. Ohodnicki

**Affiliations:** 1 Mechanical Engineering and Materials Science, University of Pittsburgh, Pittsburgh, PA 15260, USA; YAS57@pitt.edu (Y.D.S.); sunchenhu@gmail.com (C.S.); 2 Sandia National Laboratories, Albuquerque, NM 87123, USA; ypreger@sandia.gov; 3 Lawrence Livermore National Laboratory, Livermore, CA 94550, USA; burroughs5@llnl.gov; 4 Electrical and Computer Engineering, University of Pittsburgh, Pittsburgh, PA 15260, USA

**Keywords:** fiber optic sensor, fiber Bragg grating, temperature monitoring, thermal runaway, battery management systems, Li-ion battery, electric vehicle, cost estimation

## Abstract

Applications of fiber optic sensors to battery monitoring have been increasing due to the growing need of enhanced battery management systems with accurate state estimations. The goal of this review is to discuss the advancements enabling the practical implementation of battery internal parameter measurements including local temperature, strain, pressure, and refractive index for general operation, as well as the external measurements such as temperature gradients and vent gas sensing for thermal runaway imminent detection. A reasonable matching is discussed between fiber optic sensors of different range capabilities with battery systems of three levels of scales, namely electric vehicle and heavy-duty electric truck battery packs, and grid-scale battery systems. The advantages of fiber optic sensors over electrical sensors are discussed, while electrochemical stability issues of fiber-implanted batteries are critically assessed. This review also includes the estimated sensing system costs for typical fiber optic sensors and identifies the high interrogation cost as one of the limitations in their practical deployment into batteries. Finally, future perspectives are considered in the implementation of fiber optics into high-value battery applications such as grid-scale energy storage fault detection and prediction systems.

## 1. Introduction

**Figure 1:**
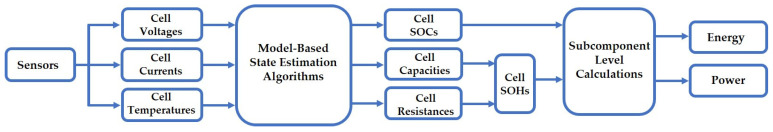
Execution flow diagram of parameter estimation algorithms involved in battery management systems (BMS) [1].

Batteries are growing increasingly promising as the next-generation energy source for power vehicles, hybrid-electric aircraft, and even grid-scale energy storage, and the development of sensing systems for enhancing capabilities of health monitoring in battery management systems (BMS) has become an urgent task. BMS play a vital role in modern electric vehicles (EVs) and other applications for battery performance management, health diagnostics, and protection against extreme conditions. As illustrated in Figure 1, these key functionalities currently rely critically on the accurate measurement of parameters such as voltage, current, and temperature as inputs to cell state-estimation algorithms. Then, model-based estimators compute the estimated capacity, internal cell resistance, and cell state-of-charge (SOC), which contribute to the further estimation of remaining energy, power, state-of-health (SOH), and state-of-life (SOL) of the battery pack [1]. Therefore, reliable and accurate input measurements are important as they affect the estimation accuracy and convergence rate of the BMS algorithms. In contemporary BMS, common temperature sensing technologies are thermocouples or micro-thermistors combined with voltage-divider circuits [1,2,3,4]. However, these sensors are based on electrical connections that may suffer from noises such as electromagnetic interference (EMI), and they cannot be integrated within the highest value locations in the cell structure. As a result, the accuracy of cell state-estimation can be limited by weakly informative parameters external to the battery, which must be extrapolated to estimate cell temperatures within each module of a battery pack. Therefore, in order to downsize and reduce the overall battery cost by optimizing the utilization of cell total capacity and preventing conservative pack design [5,6] while maintaining reliability and safety from catastrophic failures, the demand for more compact and accurate sensor technologies that can be directly deployed internal to cells has become critical.

Fiber optic (FO) sensors exhibit several key advantages over traditional electrical counterparts, which make them promising candidates to be integrated in BMS for measuring critical cell state-parameters. First, silica-based fiber optic cables are inherently immune to EMI and radio frequency interference (RFI), and they are electrically insulating [7]. Plastic fiber optic cables are also resistant to corrosive chemical species such as hydrogen fluoride (HF) that may form in Li-ion battery electrolytes [8]. Second, the light weight, flexibility, and low cost of optical fibers make it possible for them to be embedded into individual cells without significant additional size and weight, which cannot be done with conventional thermistors. Internal cell deployment can enable the monitoring of not only the internal cell temperature but also the volume expansion and phase transitions of the electrode active material that triggers capacity fade [9]. Third, the high sensitivity, multiplexing capability, and potential for functionalization to measure a wide range of parameters of interest for FO sensors allows them to detect strain, temperature, acoustic emission, and chemical species formations that serve as strong indicators of batteries’ state and health.

Based on their spatial distribution topologies (i.e., “geospatial characteristics”), FO sensors can be broadly classified into three types: single-point sensors, quasi-distributed sensors, and fully distributed sensors. A quasi-distributed sensor consists of multiple point sensors that compute measurands at fixed and discrete points along the cable, while a fully distributed sensor computes measurands along the entire region of interest of a fiber optic cable with spatial and temporal resolution dependent upon the details of the interrogation scheme. Depending on the sensing modality, physical measurands of FO sensors are typically encoded by light modulation effects such as shifts in wavelength or frequency, and changes in phase, intensity, or state of polarization. Fully distributed sensors typically require costly interrogator instrumentation to enable a large number of sensing points and therefore, they are of great interest for applications that exhibit linear characteristics such as electric powerlines, bridges, railways, tunnels, and pipelines where the value proposition is the highest [10]. The operation principle of these sensors is mainly based on light scattering including Rayleigh, Brillouin, and Raman scattering. On the other hand, in-fiber devices such as fiber Bragg grating (FBG) sensors can be used for either single point or quasi-distributed sensing and have drawn significant attention in developing enhanced battery management systems owing to two major advantages: the potential for self-referencing ability and the quasi-distributed capability to form optical fiber sensing networks through multiplexing schemes at much lower cost than fully distributed sensors [11]. In practice, common measurands of Li-ion cells such as temperature and strain are encoded by Bragg wavelength shifts, which can be expressed as a function of grating period and refractive index and their differential variation with respect to the measurands. When stimulated by these cell parameters, the FBG sensor experiences changes in its periodicity of grating and its refractive index, which then induce wavelength shifts.

FO sensors have been investigated extensively for gaseous chemical species sensing, and CO_2_ concentration has also been recently demonstrated as a crucial measurand to estimate the SOH of a battery and predict early cell failure that leads to thermal runaway. The total amount of vent gases measured before and during thermal runaway of a Li-ion battery was experimentally reported to be 2 to 4 nano-liters under thermal and mechanical treatments that simulate thermal runaways [12]. Among all reported vent gases, CO_2_ formation has been identified as the major gas generation reaction involved in solid electrolyte interface (SEI) formation and electrolyte decomposition; this degradation mechanism occurs at the electrode–electrolyte interface and drives the power fade by an increase in resistance through the deposition of decomposition products on the electrode surfaces. Not only was CO_2_ reported to account for over 70% volume fraction of all vent gases by Kumai et al. [13] and 60% to 75% volume fraction by Roth et al. [14] under abuse conditions, but it is also one of the very first generated gaseous species that can be detected before the onset of thermal runaway, which makes it the most suitable chemical for Li-ion battery health monitoring. The amount of electrolyte degradation and SEI layer formation can be inferred from the CO_2_ being measured, and then, cell SOH can be estimated by the increase in internal cell resistance due to the newly formed surface layer. Numerous other emerging CO_2_ monitoring approaches using optical fibers, such as near-infrared absorption, evanescent wave, and carbon-nanotube-coated FBG sensing, have been recently described, yielding a clear opportunity for further applications in battery monitoring moving into the future [15,16,17].

This review aims to provide a comprehensive assessment of potential applications of FO sensing technologies in battery systems with an emphasis on Li-ion batteries and perspectives for grid-scale batteries. It starts with identifying the potential applications in contemporary battery systems with different scale levels and battery types. Next, the operation principle of various types of FO sensors based on their fundamental sensing mechanism and spatial attribute topologies (i.e., “geospatial characteristics”) are reviewed, including highlights on recent trends and advances in high-performing FO sensors. This is followed by an examination on research works, within the last 5 years, in FO monitoring techniques for external and internal cell parameters, which is divided into two sections. First, monitoring strategies for general Li-ion battery functions are discussed by reviewing point temperature and strain measuring techniques for determining internal cell temperature and cell SOC, as well as other SOC, SOH correlated parameters such as pressure, acoustic emissions, transmittance, and refractive index. Second, monitoring strategies to detect imminent cell failure such as thermal runaway on a cell and pack level are discussed. Finally, a conclusion is drawn with suggestions on future developments in applying FO sensing technologies to battery systems.

## 2. Identification of Applications in Scales of Energy Storage Systems

The significant reduction in cost of Li-ion batteries has driven recent increases in the adoption of electric vehicles and stationary energy storage products. Fiber-optic sensing is currently most practical to apply on large-scale Li-ion battery products where the cost of the interrogation system can be spread across many individual battery cell or module sub-components measurement locations. A broader range of applications can become commercially viable as low-cost fiber optic sensors are commercialized in coming years. Three potential applications that we will discuss are passenger electric vehicles, heavy-duty electric trucks, and utility-scale battery energy storage.

### 2.1. Passenger Electric Vehicles

Global sales of passenger electric vehicles have been growing steadily for the last 10 years and are expected to reach over 30% of new vehicle sales by 2030, which would represent more than 30 million EVs sold in that year [18]. Growth is driven by multiple factors including decreasing cost, reduction in adoption barriers such as access to charging infrastructure, and regulations such as emissions standards. The battery size for passenger vehicles varies based on the size of the car and vehicle range. In 2020, the EV models available in the US varied from an energy capacity of 24–100 kWh [19]. Large-format EV-grade Li-ion batteries with a nominal capacity of 15 to 40 Ah have been studied using a fiber optic sensing approach at laboratory scales. [20,21,22].

### 2.2. Heavy-Duty Electric Trucks

The electric truck market is also growing quickly, with 6000 sold in 2019 [23]. The market is expected to expand due to both decreasing costs and regulations such as the California Air Resources Board zero-emission truck mandate [24]. Electric trucks can vary in size from light-duty delivery trucks to heavy-duty long-haul trucks. For the largest electric trucks on the market, the battery pack size varies from about 300 kWh to 1 MWh [25,26,27]. 

### 2.3. Utility-Scale Battery Systems

**Figure 2:**
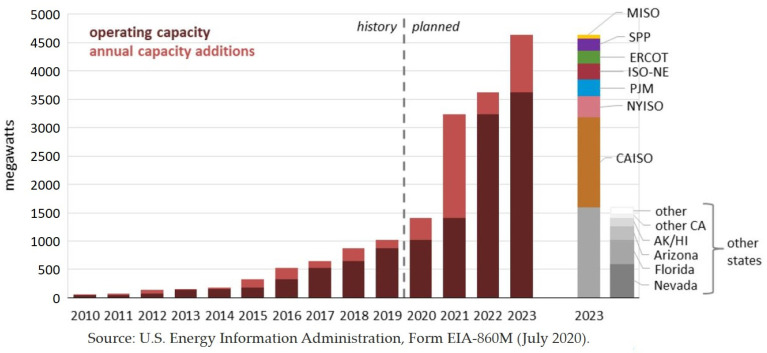
Large-scale battery storage cumulative power capacity (2010–2023) [28].

As the composition of generation sources on the grid diversifies and becomes more renewable, the application for and installation of utility-scale battery energy storages systems is rapidly increasing, and the size of individual sites is also growing. Figure 2 from the U.S. Energy Information Administration reports that planned large-scale battery storage in the US will quadruple in the next three years [28]. Part of what is driving the growth in installed capacity is the increasing size in the largest sites. In the last five years, the scale of the world’s largest individual battery installations has grown from single MWhs to 100 s of MWhs [29]. 

### 2.4. Comparison between Use Cases and Sensors

Table 1 provides a detailed comparison between the number of cells needed for electric vehicle and grid-scale Li-ion based energy storage systems. There are three commonly used cell constructions: cylindrical cells (such as the 18650), prismatic cells, and pouch cells [30]. Depending on the cell type and application, hundreds to millions of cells may be required.

These individual cells are electrically connected to make larger subcomponents, which are sometimes referred to as modules or packs. The number of cells in parallel and series used to create these subcomponents depends on a specific manufacturer’s design with factors considered including the desired voltage range for the power electronics and consideration of the desired relationship between maximum power and energy capacity. For vehicle batteries, the dimensions of the vehicle dictate the overall size and shape of the battery pack. For grid-scale batteries, two common units (beyond the module) are a shipping container-sized unit, such as the NEC Energy Solutions 20 ft, 40 ft, and 53 ft container options [37] (with walkable hallway and racks of batteries) or a smaller custom-made pack such as the Tesla Powerpack (4 ft × 3 ft × 7 ft) [38] or the Fluence Cube (10 ft cube) [39]. Then, these modular units are duplicated as many times as needed to build a utility-scale site.

To maintain both safety and longevity of a Li-ion battery system requires careful monitoring of cells and adjustment of the operating behavior (rate of charge/discharge and thermal management) as a function of the cells’ current conditions. The key physical characteristics typically monitored by a BMS are the battery cell (or set of parallel cells) voltage, the cell (or set of series cells) current, and the module or ambient air temperature. Cell-level temperature monitoring is not typical in contemporary electric vehicle or battery storage applications due to the high cost of individual cell monitoring and the typically small difference in temperature variance between cells that are in the same operating conditions. However, in the case of abnormal behavior such as internal damage to a cell, the damaged cell’s temperature would deviate from its neighbors. While this type of event is rare, the worst-case consequence is thermal runaway.

The recent Li-ion utility-scale battery fires in South Korea and Arizona [40] have led to increased interest in technologies for early thermal runaway detection. Thermal runaway involves a positive feedback loop between heat generation and the degradation of battery components. Various abuse conditions can trigger Li-ion battery thermal runaway: a short circuit (from a defect within the cell), over charge or discharge, physical damage, or external heating [41]. Without adequate detection or mitigation, thermal runaway in a single cell may propagate to other cells or modules. Consequently, there is great interest in monitoring technologies that can detect warning signs of thermal runaway early enough to avoid it entirely (for example, by preventing further charging of a damaged cell) or at least prevent propagation. More accurate and robust sensing technologies must detect characteristic features of thermal runaway such as a voltage and current anomaly, a local temperature rise, or a gas venting phenomenon, as discussed in more detail in the following sections.

A common limitation of conventional sensors that can be overcome by FO sensors is the inaccessibility into compact and corrosive environments. Take temperature sensors as an example; Table 2 is adapted from Raijmakers et al. [42] with additions and enhancements, and it provides the criteria to compare general FO sensors with conventional temperature sensing methods for Li-ion batteries. The cost information provided here is illustrated by the fixed total base cost (from representative vendor quotes at the time of publication) and the cost for a single sensing point that could be significantly lowered by incorporating more sensor elements as the application scales up and multiple sensor elements are integrated with a single interrogator system. Examples of potential costs per FBG sensor node possible for different scales of batteries with multiple gratings for a single fiber are also presented in Table 2. This assumes that for each application, one FBG sensor monitors every five Li-ion large-format pouch cells with the cell dimension and total number of cells taken from Table 1. It is assumed that the cost per sensor is $10/FBG, and the single mode fiber cost is $3/meter. Therefore, the estimated cost per sensing point can be calculated as shown below.

(1) $$\frac{Interrogation\;Fixed\;Cost\;+\;\#\;of\;cells\times 1\;\frac{FBG}{cell}\times \frac{\$10}{FBG}\;+\;Total\;Length\;of\;Fiber\times \;\frac{\$3}{meter}}{\#\;of\;sensor\;elements}$$

The example of total sensing system costs based on the equation above are $10,725, $15,500, and $1,100,000 for EV, electric truck, and grid-scale energy storage applications, respectively. The total estimated sensing system cost for an EV is nearly 1/4 of the price of the vehicle itself. This result shows that the dominating interrogation cost and cell-level implementation can make it impractical for FBG sensors to be commercially used in battery packs of EVs. Less expensive interrogation/demodulation tools need to be developed to justify the commercial use of FO sensors in contemporary battery applications. In addition, more attention needs to be drawn to other low-cost FO point sensors rather than only on FBG sensors. Various types of FO sensors are comprehensively discussed in the next section.

## 3. Operating Principle and Recent Advances in Fiber Optic Sensors

Interest and efforts have grown in the past five years in the experimental validation of implementing FO sensors in Li-ion batteries to explore the feasibility and address the need of battery systems for more powerful sensing technologies. Before diving into the details of specific research cases, this section provides a concise review on the fundamental principles and recent developmental trends of common types of FO sensors. This includes both point sensors and distributed sensors that are of potential and existing interest to the battery community.

### 3.1. Single-Point Sensors

#### 3.1.1. Fiber Bragg Grating Sensors

FBG sensors are based on periodic modulations of refractive index along the length of the fiber core, and these perturbations are known as fiber Bragg gratings. When the incident light propagating in the optical fiber interferes with the back-reflected light from interfacial discontinuities, a standing wave pattern is formed and inscribes index gratings into the fiber through the photosensitivity effect: a permanent change in the refractive index of a fiber due to ultraviolet (UV) radiation [53]. Detailed expressions of mode-coupling between reflection and transmission spectrum are illustrated in [54]. The Bragg wavelength $\lambda_{B}$, at which the strongest interaction and coupling of incident light and its counterpart occurs, can be defined as:

(2) $$\lambda_{B}=\;2\;n_{\mathrm{eff}}\Lambda ,$$

where $n_{eff}$ is the effective refractive index and *Λ* is the grating period. The shift in Bragg wavelength is sensitive to fiber properties that vary with the refractive index or grating period of the fiber; therefore, it serves as a strong indicator of changes of the environmental parameters external to the fiber. Possible parameters include temperature, strain, chemical concentration, pressure, acoustic emission, and refractive index. The fabrication techniques of FBGs can be categorized into two types: phase mask and mask-less techniques. The phase mask technique is usually an interferometric method that defines the grating period by illuminating a corrugated mask made from silica glass through UV light; mask-less techniques are the point-by-point method and continuous core-scanning method, in which the grating is written by controlling the translational movement of the fiber [53,54].

**Figure 3:**
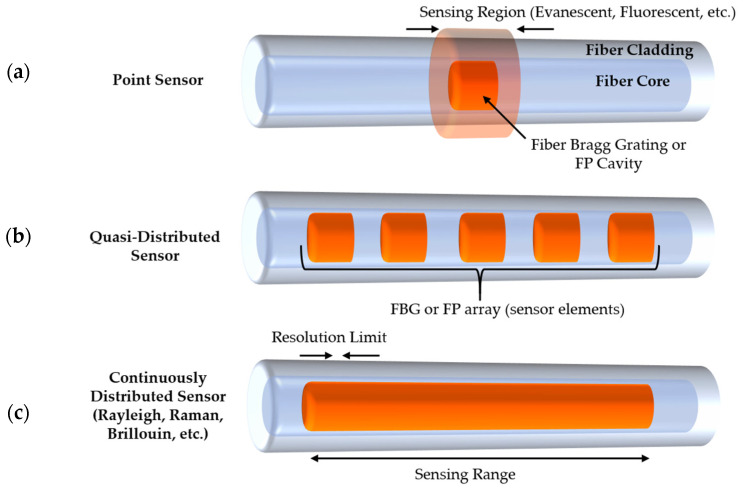
Schematic representation of the key components in (**a**) single-point, (**b**) quasi-distributed, and (**c**) continuously distributed fiber optic sensors.

Other than equally spaced gratings with a uniform Bragg wavelength and modulation strength, FBG sensors can be optimized for a particular sensing objective by phase-shifting or chirping the fiber gratings with varying grating period or by apodizing the grating profile with non-uniform index modulation strength [54]. Chirped FBG has a broadened reflection bandwidth and enhanced spectral response with reflection spectrum expressed as a function of position along the grating. As the correlation between battery SOC and acoustic intensity has been previously demonstrated, extensive research in developing FBG-based sensors as a sensing element for acoustic emission measurements exhibits great potential in determining the SOC of a battery. Specifically, Hu et al. demonstrated the use of π-phase shifted FBG as a promising acoustic emission sensing method owing to its noise cancellation capability [55]. The reason is that the acoustic signal strength, which is proportional to the spectral slope of the reflection spectrum of the fiber gratings, can be enhanced by the steep spectral slope of the π-phase shifted FBG. The signal-to-noise ratio (SNR) was reported to increase by 20 dB using this method. Key performance factors that characterize an FBG as well as a typical FO sensor include the sensitivity, resolution, bandwidth, and accuracy of the measurement [54]. Figure 3a gives a conceptual overview of four common types of FO single-point sensors.

#### 3.1.2. Evanescent Wave Sensors

Fiber optic evanescent wave (FOEW) sensors are typically based on an optical fiber core with semi-absorbing cladding materials coated on the surface, with an absorption coefficient of the cladding dependent upon the analyte of interest. Evanescent waves (EW) are formed when incident electromagnetic light propagating through the fiber core undergoes total internal reflection at the core–clad interface, where the light strikes at an angle greater than the critical angle [56]. The evanescent wave experiences exponential decays in its field strength as a function of propagation distance from the core surface into the surrounding medium through absorption, corresponding to a transmittance loss in the electromagnetic energy of the guided wave when passing through the sensing region. These losses can be measured as a function of surrounding temperature, atmospheric chemical species, or other parameters interacting with the sensor. The exponentially decaying evanescent field is able to interact with materials coated on the sensing region. The evanescent wave penetration depth, $d_{p}$, into the surrounding medium is defined as [57]:

(3) dp= λ2π(nco2 sin2θ−ncl2)12 ,

where the incident light with a wavelength of *λ* is internally reflected at an angle *θ* at the interface of the core and the cladding with refractive index of $n_{co}$ and $n_{cl}$, respectively, assuming the imaginary optical constant can be neglected relative to the real values. The performance metrics, such as sensitivity and resolution, of FOEW sensors depends on the evanescent wave absorption coefficient and the penetration depth in the sensing region, which also depends upon the sensing layer coating as well as the geometry of the sensor probe. Over the years, numerous techniques such as U-shaped, tapered, and D-shaped FOEW sensors have been developed to enhance the evanescent field and increase the penetration depth of the EW interacting with the analyte [58,59,60]. Sharma et al. have summarized a detailed review on the important advancements of FOEW sensors in terms of geometries and coating materials in the recent decade [56]. Among FOEW sensors, D-shaped plastic fibers have been successfully simulated and identified as a particularly practical design because of relatively low cost and simple fabrication while retaining the effect of enhancing sensitivity through controlling the curvature radius, depth, and length of the grooved region [61]. An additional technique described in the literature involves the optimization of the absolute refractive index of the sensing layer through engineered porosity as in block co-polymer-based templating techniques [62,63,64]. FOEW sensors based on infrared (IR) glasses introduce a wider optical transmission window against conventional silica-based fibers from 2 µm up to ≈25 μm, but they are typically expensive, which limits their deployment in cost-sensitive applications [56]. Nevertheless, Maurugeon et al. reported selenium-modified GeTe4 based FOEW sensors to increase the accuracy of detecting the broad absorption band of CO_2_ at 15 μm [65], which has great potential in SOH estimation and early failure detection of Li-ion batteries based on CO_2_ sensing. To improve the EW absorption response of gaseous chemical sensing without the cost of integrating FBGs or the usage of expensive mid-IR or IR fibers, novel materials such as Au-nanoparticle based plasmonic nanocomposite thin films, polymers, and metal–organic frameworks have also been demonstrated as the sensing layer for FOEW sensors at both ambient and elevated temperatures [66,67,68,69,70,71,72]. 

#### 3.1.3. Fluorescence-Based Sensors

Fluorescence is a form of luminescence emitted by a fluorophore that has absorbed electromagnetic radiation. Employing fluorescence spectroscopy in FO sensors offers the advantage of higher intrinsic sensitivity over absorbance as well as the flexibility and versatility of fluorophores to interact with a variety of analytes. Signal changes include intensity, lifetime, color, wavelength, and polarization of emission. For example, lifetime measurements can be done by measuring the sinusoidal phase shift between exciting radiation and the fluorescence emission [73]. Luminescence-lifetime-based FO gas sensors have been extensively developed, particularly for O_2_ sensing [74], making it a promising candidate to be deployed in a Li-ion battery system, as oxygen is a possible vent gas during SEI decomposition [41]. Two major fluorescence techniques being applied are anisotropy decay and quenching. The former depends on the time-dependent orientation of the fluorophores, while the many mechanisms of the latter can be described by the Stern–Volmer equation [75]. In [76], Yang et al. categorized the arrangement of fluorescence fiber optic sensing into extrinsic and intrinsic sensing schemes. Extrinsic fluorescence fiber optic sensors require one optical fiber to guide the incident light wave and excite the fluorophore near the fiber, and another fiber to collect the emitted fluorescence radiation. This approach is often applied in optical pH fluorometers and metal cations sensors in aqueous solution using pH or cation sensitive membranes.

On the other hand, in intrinsic fluorescence fiber optic sensors, the excitation and reception of the fluorescence are carried out in one single fiber, with additional optical requirements to develop a suitable optical configuration to separate excitation and emission based on the wavelength difference. In the case of intrinsic sensing schemes, the evanescent wave is often used to excite the fluorescent compound for triggering fluorescent emission owing to the advantage that the fluorescence intensity can be enhanced by reducing interference from bulk solution and that it only excites the fluorescent materials close to the fiber [75]. In battery system monitoring, fluorescent fibers have been embedded in Li-ion batteries as a sensing probe to monitor internal cell temperature by Du et al. [41,77]. In this work, the temperature is measured based on a negative correlation between temperature and the decay of lifetime of the received fluorescence radiation from the optical fiber. Other than point temperature measurements, area-based fluorescence imaging such as fluorescence microscopy was recently used for research purposes in Li-ion batteries to track the ionic transport in electrolyte and electrode structures. Padilla et al. demonstrated a detection method using fluorescence microscopy based on widefield imaging with cation-sensitive fluorophores to quantitatively determine the diffusion constant of lithium ions [78]. However, further investigations are required into the electrochemical stability of this lithium-ion sensitive fluorescent indicator.

#### 3.1.4. Fabry–Perot Interferometer

An optical fiber Fabry–Perot (FP) interferometer is a point sensor characterized by the optical cavity created between two parallel reflective surfaces by the separation distance, which consists of fiber–cavity interfaces [79]. In the cavity, superposition of the light waves occurs when the propagating light is transmitted and reflected multiple times between the interfaces, which then results in their interference with each other. The intensity modulation spectrum is caused by the optical phase difference between two reflected or transmitted light waves [80]. The phase variation is related to the variation in the optical path length difference of the interferometer [80]. The length of the cavity and the refractive index of the cavity material vary as the fiber experience external perturbations such as strain and temperature, which then changes the optical phase. These measurands can be quantified by measuring the wavelength shift in the intensity spectrum. The wavelength-dependent phase difference, $\delta_{FP}$, of an FP sensor cavity can be defined as:

(4) $$\delta_{FP}=\;\frac{2\pi }{\lambda }\;n2L,$$

where *n* is the refractive index of the cavity material, *L* is the distance between two reflectors (length of the cavity), and *λ* is the wavelength of the incident light. Depending on the formation and position of the cavity, optical fiber FP sensors can be categorized into extrinsic and intrinsic sensors. Extrinsic FP sensors have the cavity built on the outside of the fiber, which is suitable for monitoring liquids, as the cavity can be easily accessed by the measurands. The refractive index of the liquid has a positive correlation with the optical path length difference of the cavity, which can be observed by locating the Fourier peaks from taking the Fourier transform of the reflection spectrum, which is described in detail by Lee et al. in [80]. Low coupling efficiency has been reported to be a disadvantage of extrinsic FP sensors [81], but it can be solved through strategies such as the addition of a photonic crystal fiber and a fiber lens. Extrinsic FP sensors can also be implemented as displacement, pressure, and acoustic sensors by applying thin film polymers, which are deformation-sensitive, to the surface of the external reflectors [82,83,84]. This is due to the lower Young’s modulus of the applied polymer as compared to the cavity material. 

Intrinsic FP sensors, on the other hand, have the reflectors formed on the inside where the cavity material is usually the fiber itself. One major advantage of intrinsic FP sensors is that the inner cavity is free from the disturbance of the potentially harsh chemical environment on the outside, which might otherwise cause measurement errors. A double cavity fiber FP sensor is a unique example of the intrinsic FP sensors with two cavities created by a holey optical fiber fusion spliced between a single mode and a multi-mode optical fiber. As a result, the Fourier spectrum of this type of FP sensor is characterized by three major peaks: the ones caused by the first and second cavities, and by the combination of both [80]. A double cavity FP sensor can not only serve as a temperature sensor with the determination of different thermo-optic coefficients of the inner cavities but can also be implemented as a gas sensor through surface coating of a chemically sensitive material on the end interface of the multi-mode cavity. Intrinsic FP sensors have been demonstrated in battery system temperature monitoring both for research purposes and commercial products [47,85]. 

### 3.2. Distributed Fiber Optic Sensors

#### 3.2.1. Quasi-Distributed Sensors

Quasi-distributed sensors, as illustrated in Figure 3b, are discrete sensors enabled by the multiplexing capability of point sensors that could increase the span of the sensing area for measuring the same or different measurands along the fiber optic sensing network. The number of sensor elements that can be deployed on a single fiber depends on the multiplexing schemes being used. These methods include wavelength-division multiplexing (WDM) [86,87], time-division multiplexing (TDM) [88,89], frequency-division multiplexing (FDM) [90], and code-division multiplexing (CDM) [91]. Among these, WDM and TDM are two of the most common techniques to multiplex FBG sensors. While the former relies on the difference in Bragg wavelength that can be reflected by each FBG sensor element using a multi-wavelength light source, the latter utilizes gratings with the same wavelength tuned by a pulsed laser source and the resulting difference in time delays of the return reflections on the photodetector [92]. In a WDM sensing scheme, the maximum number of sensor elements, which is limited by the ratio of the system bandwidth to the dynamic wavelength range of an individual FBG sensor, is usually tens of FBGs [93]. As opposed to WDM, TDM can incorporate a great number of measurable sensor elements, as many as 1000, by developing new network configurations to differentiate the delay time of each element [93]. 

Two major TDM topologies are serial and parallel configurations, where a trade-off exists between system complexity and multiplexing capacity. To improve performance without the cost of a complicated topology using parallel TDM networks, past efforts have been put particularly on a serial TDM multiplexing scheme [93,94,95,96], where researchers investigated the theoretical background and application of TDM on FBG sensors. A quasi-distributed FBG sensor based on resonance frequency mapping, which is induced by the total cavity lengths determined by the positions of multiple FBGs with respect to one another, was proposed recently by Kim et al. [97]; they addressed issues such as low SNR, high crosstalk, and optical power loss. Quasi-distributed FBGs can also be integrated with fiber optic interferometers. Fabry–Perot, Mach–Zehnder, Michelson, and Sagnac interferometers have been demonstrated to have multiplexing capabilities [91,98,99,100] and reported with numerous configurations including matching a uniform pair of FBGs or long-period gratings (LPG) to enhance the sensitivity to a measurand [92,101]. The integration of Fabry–Perot and FBG sensors is a novel approach to discriminate and simultaneously measure the strain and temperature responses [102,103], which is a critical task in the internal parameter monitoring of battery systems and will be discussed in detail in Section 4.2.

Recent advances in this field involve the implementation of an in-line multiplexed interferometric structure with two optical paths in a single fiber to minimize the size of fiber optic sensors [80,104]. To apply quasi-distributed sensors in energy storage applications, one key aspect is to accurately match the scale of the device with the most feasible multiplexing technique that would generate the highest value proposition. The details of proposed solutions are presented in Table 3. For example, in a grid-scale battery pack of 100 MWh, a carefully configured TDM may be required to accommodate 100 s to 1000 s of sensing points for the detection of abnormal thermal events through distributed measurements; for applications such as battery packs in electric aircrafts and long-haul trucks, a WDM/CDM or WDM/FDM combined method can be used for tens to 100 s of sensors; on the other hand, in battery packs for passenger EVs, a conventional WDM might be enough to deploy distributed temperature gradient sensing or thermal runaway detection with tens of FBG sensor elements at the module level.

#### 3.2.2. Continuously Distributed Sensors

Fully distributed fiber optic sensors (DFOS), as illustrated in Figure 3c, are continuous sensors that have the advantage of high sensing capacity for large-scale monitoring in temperature, strain, and gas distributions. Their working principles are mainly based on light scattering in the form of Rayleigh, Brillouin, and Raman scattering. The detailed physics background of these scattering phenomena is thoroughly reviewed in Lu et al.’s work [10]. In short, Rayleigh scattering originates from the refractive index change due to density fluctuations in optical fibers, where the frequency of the incident wave does not change as it scatters. Brillouin scattering, on the other hand, results from scattering from acoustic phonons created by a thermally induced acoustic wave, which then leads to a frequency shift of the scattering wave in the fiber due to the inelastic photon–phonon interactions. Similarly, Raman scattering is inelastic in the sense that it generates a frequency shift between the pump photons and the scattered photons that results from molecular vibrations. Two major interrogation techniques for DFOS are optical time-domain reflectometry (OTDR) and optical frequency-domain reflectometry (OFDR). OTDR requires a high-power light pulse traveling along the fiber and creates a backscattered pulse in which the signal is a function of the detection time at the photodetector. The sensitivity and SNR of an OTDR system increases with the pulse power, pulse duration, and photodiode sensitivity; however, a trade-off exists between the sensitivity and spatial resolution of OTDR due to the width change in pulse duration [105]. In Rayleigh-scattering-based DFOS, conventional OTDR can be extended to polarization-sensitive OTDR and phase-sensitive OTDR with the former measuring the spatially resolved state of polarization of the backscattered light and the latter utilizing a laser pulse with a coherence length longer than the fiber’s length to enable interferometric measurements based upon measured phase shifts [106,107]. Even though Brillouin-based OTDR sensing has been identified as the most widespread system in energy infrastructure structural health monitoring [10], it is not discussed in the current article because it is most valuable when the sensor distribution distance is up to tens of kilometers, which is not relevant for energy storage applications where cost reduction is the determining factor.

OFDR has been an attractive candidate for distributed temperature sensing (DTS) owing to its relatively higher spatial resolution, which is a function of the optical frequency sweep of a continuous wave light source, as compared to traditional OTDR systems [10]. With different types of light sources, coherent OFDR relies on modulation of the frequency of coherent light, while incoherent OFDR depends on modulation of laser intensity. Vergori et al. has demonstrated the monitoring of distributed temperature and strain gradients using Rayleigh scattering based OFDR sensors in a cycling Li-ion pouch cell [108]. The stable result in this work eliminates the potential error in point sensing with pre-defined locations and highlights the possibility of locating abnormal cracks and temperature hotspots due to cell aging. Rayleigh-based OFDR systems have also been applied to the temperature monitoring of the magnetic core and insulation oil of a power transformer by Lu et al. [109] and Badar et al. [110,111]. The above cases have confirmed the unique advantage of immunity to EMI of fiber optic sensors in high power electrical components and demonstrated their distributed sensing capability, which is highly critical to monitor the health of these electrical assets. Another DTS techniques that has been widely used is Raman-based OTDR [112]. Recent advances in this field have been focusing on producing larger Raman signals by increasing the allowable stimulated Raman scattering threshold that limits the maximum laser power required to generate sufficient Raman photons for measurement [10,113]. Despite all the advantages of DFOS in large-scale energy storage systems and in battery failure detection, DFOS suffer from some drawbacks such as ultraweak backscattering signals within fibers, higher cost, and interrogator system complexity relative to quasi-distributed FO sensors [97]. Further analysis needs to be done in comparing the economic feasibility and technical benefits of quasi- and fully distributed sensors to evaluate potential applications in commercial grid-scale energy storage systems.

## 4. Parameter Monitoring for General Operation of Batteries

To manage the safety and functionality of batteries, it is important to track internal cell-state variables such as SOC and SOH during operation. Errors and uncertainties in SOC estimation can result in abuse charging, conservative battery pack design, and extra weight added to the system to meet energy demand, while those associated with SOH can lead to thermal events and accelerated aging within the cells. The potential advantage of increasing the estimation accuracy for these states has been investigated with the use of FO sensors. Several parameters measured by FO sensors show correlation with cell internal states. These measurands include local temperatures and strain, both at the surface and internal to the cell, pressure, refractive index, and transmittance. This section highlights the recent efforts involved in demonstrating the measurement of these parameters for battery general operation management. Advancements as well as current limitations are discussed.

### 4.1. Point Temperature Measurements

Thermal management has been a critical part in battery system design both in the aspect of general cell operation and cell degradation rates control. One important temperature-dependent cell operational characteristic is open-circuit-voltage (OCV). As OCVs are widely used in equivalent-circuit-model-based SOC estimation algorithms in contemporary BMS [1], the reliability of estimated SOCs demands accurate point measurement of the temperature that is representative of an entire cell. In a recent work, in order to monitor the cell temperature stably without damaging a bare fiber, Peng et al. [114] proposed a FBG sensor configuration with protective metal rings and covers to capture the temperature variations of a lithium iron phosphate (LFP) battery during standard charge/discharge cycles. The proposed FBG was placed on the tabs of the pouch cell, and the resulting temperature profile was verified by a commercial resistance temperature detector (RTD) and exhibited good agreement. However, the lack of further investigation into internal cell temperature can become an issue due to the fact that the difference between the external and internal temperature of a Li-ion battery can reach as high as 20 °C under large current rates, as reported in several thermal characterization simulations and laboratory studies for EV batteries [115,116,117]. 

**Figure 4:**
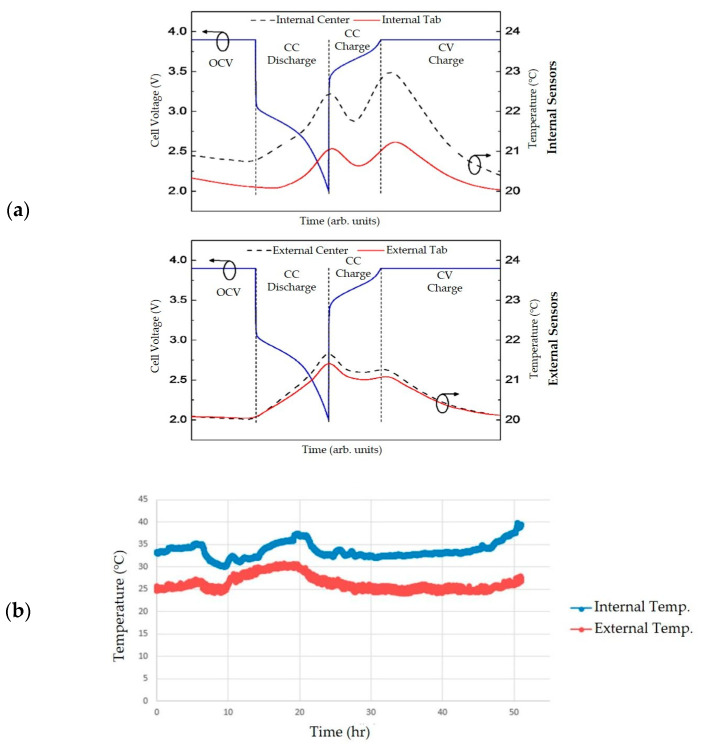
External vs. internal cell temperature difference by fiber Bragg grating (FBG) sensors: (**a**) temperature change from internal and external measurements of Li-ion pouch cell under 5 C-rate discharge/charge [118]; (**b**) internal cell and ambient temperature data of Li-ion coin cell under C/20 rate [119].

To address this issue, Novais et al. [118] demonstrated a method for embedding optical fibers with FBG sensors placed at the center of the active materials and near the tab-electrode connection area in a self-assembled LFP pouch cell. These internal measurements were compared to another set of FBGs at the corresponding positions on the cell surface. A clear difference was observed in the temperature variation of the tab-electrode connection and center area measured by the internal and external FBGs. Figure 4a shows a 2 °C temperature variation between these two areas measured by the internal sensor and only a 0.2 °C difference by the external sensor. While this study achieved a successful integration of FO sensors into batteries and internal temperature measurements, it perhaps incorrectly assumed that the strain changes due to cell volume expansion are negligible. Since strain variations can also be detected in an FBG, this assumption may result in an overestimation of the cell internal temperature as the strain-induced wavelength shifts are falsely attributed to temperature.

A more detailed study of both strain and internal cell temperature responses was conducted by Fortier et al. [119]. Li-ion coin cells were studied with a slotted location for FBG sensor probe insertion created on the side and with appropriate sealant material. Four separator layers were wrapped around the optical fiber to prevent it from cutting through the cathode active material. The results highlighted the strain changes within the cell and a prominent difference of 10 °C, as shown in Figure 4b, between the external and internal cell temperature for 50 h at the C/20 rate. Critical battery geometric challenges for sensor integration were discussed, and recommendations were given for long-term monitoring of hermetically sealed cell. However, careful assessment is again needed regarding the discrimination between strain and temperature-induced signals.

### 4.2. Strain-Temperature Discrimination Methods

**Figure 5:**
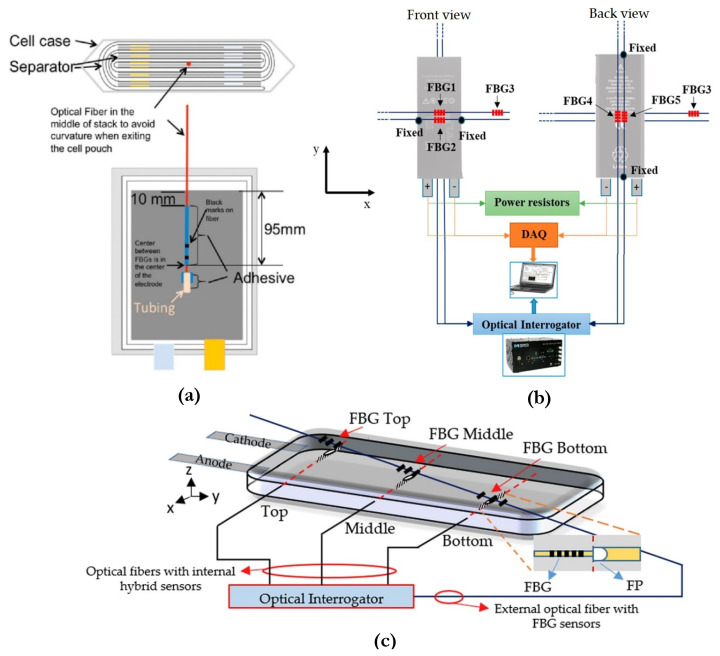
Schematics of various strain-temperature discrimination methods: (**a**) embedded FBGs in an Li-ion pouch cell using the reference sensor method (Reproduced with permission from Raghavan et al., Journal of Power Sources; published by Elsevier, 2017.) [21]; (**b**) experimental setup of externally attached FBGs on an Li-ion pouch cell surface using the reference sensor method [121]; (**c**) externally attached hybrid FBG–Fabry–Perot (FP) sensors on an Li-ion pouch cell surface using a single fiber discrimination method (Reproduced with permission from Nascimento et al. Journal of Power Sources; published by Elsevier, 2019.) [85].

One major challenge in the demodulation of FBG sensor signals is that the Bragg wavelength shift, $\lambda_{B}$, is a complex convolution of several physical parameters including temperature, pressure, and strain, and therefore, it suffers from cross-sensitivity [120]. Among these, strain (ε) and temperature (T) are of most interest in battery systems, and the contributions of both to Equation (2) can be described as follows:

(5) $$\frac{\Delta \lambda_{B}}{\lambda_{B}}=\left(\frac{1}{\Lambda }\;\frac{\partial \Lambda }{\partial T}+\frac{1}{\;n_{eff}}\;\frac{\partial \;n_{eff}}{\partial T}\right)\Delta T+\;\left(1-\;\rho_{e}\right)\;\Delta \varepsilon ,$$

(6) $$\rho_{e}=\;\;n_{eff}{\;}^{2}\left[\left(1-\mu \right)p_{12}-\;\mu p_{11}\right]/2,$$

where *Λ* is the grating period, $\;n_{eff}$ is the effective refractive index, $\rho_{e}$ is the effective photo-elastic coefficient of the fiber core, μ is Poisson’s ratio and $p_{11}$ and $p_{12}$ are the Pockel’s coefficients of the strain optic tensor [11]. A discrimination method to decipher these parameters is proposed by Raghavan et al. [21], where the internal strain and temperature of a Li-ion pouch cell are measured and further applied to SOC and SOH estimators. Similar to Novais et al. [118], Raghavan et al. used a protective heat seal material to ensure the hermeticity of the cell and placed the FBG fiber probes at the most informative center area of the cell in between two separators. One of the fibers was mechanically decoupled from the cell internal structure by enclosing it in a heat-conductive tube. Then, this enclosed FBG sensor served as a reference that is assumed to be insensitive to strain but does respond to temperature changes. Thus, the individual contributions of temperature and strain to wavelength shifts can be separated by subtracting one from another and by deriving a temperature compensation factor either experimentally or analytically using a statistical algorithm, as elaborated in [21]. Similarly, Nascimento et al. [121] discriminated strain and temperature effects within a Li-ion prismatic cell by fixing one FBG fiber with thermal paste and leaving the other unfixed, which can be considered as a reference sensor that only detects temperature changes. Figure 5a,b give the schematic view of this reference sensor method by two research groups. 

Other than using two different FBG fibers, discrimination methods based on different grating sensor elements on a single optical fiber are also presented [122,123], however, with a concern of limited spatial resolution. To address this issue, Nascimento et al. [85] then introduced an improved method that combined the signals of FBG and the FP sensor by inscribing the FBG as close as possible to the FP air cavity, based on the fact that FP sensors with high air cavities are highly sensitive to strain variations. As can be seen in Figure 5c, the cavity was created by fusion-splicing an FBG-written single mode fiber (SMF) and a multi-mode fiber (MMF). The changes in both strain and temperature can be solved by two wavelength formulas from FBG and FP, given the pre-determined sensitivity rate constants. Three sensing points were placed from top to bottom of a smart phone pouch cell, and the middle position was observed to exhibit the most significant changes in both parameters of interest. This method of simultaneously measuring two parameters using a single fiber of two types of FO sensor elements eliminates the need of tubing to create a strain-free reference sensor and the need of using a pair of fibers, which would otherwise increase the intrusiveness and potentially damage the capacity of the electrochemical active materials.

### 4.3. Parameters for SOC and SOH Estimations

#### 4.3.1. Strain Measurements 

Strains are a result of stresses generated from the inside of an Li-ion battery. Important stress factors include volume expansions associated with intercalation at both electrodes, SEI formation at the anode, phase transitions of the cathode, and thermal expansions/contractions due to internal resistive heating and electrochemical reactions [11]. When the strain and temperature-induced signals are separated, the resulting strain measurements have been shown to have high correlation with both SOC and SOH of Li-ion batteries, and therefore, extended cycling data at a range of different C-rates can serve as training datasets to build an SOC or SOH estimation model. Experimental approaches to implement a strain measurement by battery research groups can be broadly classified into external measurements and internal measurements. Sommer et al. [9] deployed a strain measurement from the surface of an NMC-based Li-ion pouch cells with the reference sensor method and identified the intercalation stage transition points via peaks generated in the strain-induced wavelength signal spectrum. Not only was the elevated strain due to electrode volume expansion observed during the charging phase, but the residual strain due to excess volume change in the outer region of the electrode active materials and the strain relaxation at the subsequent rest phase was observed at 80% to 100% SOCs in the team’s following work [124]. These strain fluctuations at high SOCs, resulting from inhomogeneous lithium ion diffusion between the inner and outer region of the active materials, are a key cell failure mode that is a source of capacity fade. Therefore, incorporating excess strain-triggered signals into BMS to adjust charging profiles can help optimize cell performance and lifetime. Another strain factor is confirmed by the signal discrimination work using hybrid sensors by Nascimento et al. [85], where the strain resulting from thermal expansions and contractions was validated by comparing the separated temperature and strain signals.

**Figure 6:**
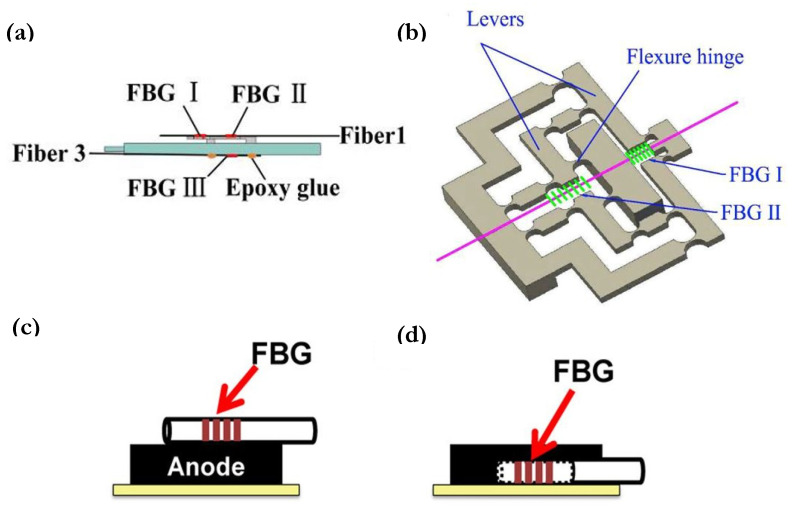
Schematics of strain monitoring strategies: (**a**) externally attached FBG strain sensor on an Li-ion pouch cell with enhanced sensitivity (Reproduced with permission from Peng et al., Journal of Power Sources; published by Elsevier, 2019.) [125]; (**b**) sensitivity-enhancing structure (Reproduced with permission from Peng et al., Journal of Power Sources; published by Elsevier, 2019.) [125]; (**c**) internally attached FBG strain sensor on graphite anode (Reproduced with permission from Raghavan et al., Energy Technology (Electronics); published by John Wiley and Sons, 2016.) [126]; (**d**) internally implanted FBG strain sensor within graphite anode (Reproduced with permission from Raghavan et al., Energy Technology (Electronics); published by John Wiley and Sons, 2016.) [126].

Other than local point measures, external strain sensing has also been done by quasi-distributed FBGs that monitored the strain evolutions of a 96-cell EV battery pack [20] and by a Rayleigh-scattering-based distributed FO sensor that studied the strain gradient across the width of a cell [108]. As most previous FBG strain sensor studies were done with bare fibers that may suffer from low strain sensitivity and instability for long-term monitoring, Peng et al. [125] proposed a strain-sensitivity enhancing protection structure, as shown in Figure 6a,b, for the FBG fiber attached external to the cell. This structure was based on the theory of strain concentration and lever amplification. The mechanical properties of the silica fiber and the steel protection were optimized through finite element analysis and achieved an 11.69 times greater strain-induced signal response.

**Figure 7:**
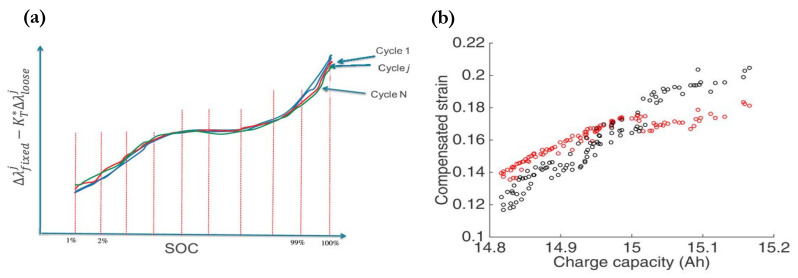
Experimental data of strain versus state-of-charge (SOC) and charge capacity of Li-ion pouch cells [22]: (**a**) correlation identified between temperature-compensated strain (vertical axis) and SOC; (**b**) variation of strain with charge capacity. (Reproduced with permission from Ganguli et al., Journal of Power Sources; published by Elsevier, 2017.).

For internal strain measurements based on FBGs, Bae et al. [126] conducted a comparison between positioning the fiber central to both electrodes and embedding it into the anode active materials, where conventional electrical-based strain gauges cannot reach. Figure 6c,d give a schematic view of the two cases respectively. The positive correlation between strain and SOC was observed as well as a four-times higher strain evolution than the centered reference FBG by the implanted FBG at 100% SOC. The same strain and SOC relationship was observed by Ganguli et al. [22], a study in which the compensated strain versus SOC data points under a range of C-rates were used to train an SOC estimator based on a dynamic time warping algorithm. An estimated SOC accuracy of over 99% was reached for the static cycle testing, while for dynamic cycle test data, a more sophisticated algorithm such as Extended Kalman filter (EKF) was used. The strong relationship between strain and capacity was also presented, and the training data of end-of-charge maximum strain versus charge capacity was used to construct an SOH estimation algorithm based on the predicted cell capacity. Figure 7 shows examples of the training data of these correlated variables. As previously mentioned, the estimation of SOH is a coupling between capacity fade and power fade from an increase in cell internal resistance [1]. Even though the SOH model by Ganguli et al. failed to consider the effect of resistance, the work demonstrated promising results of FO sensors for improved state-parameter estimation in BMS over conventional sensors.

#### 4.3.2. Other Parameters for SOC and SOH Estimations

**Figure 8:**
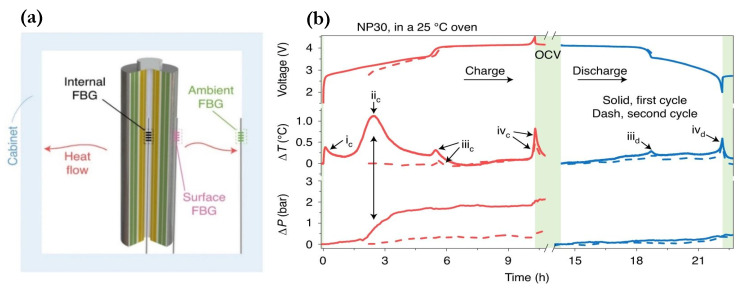
Pressure and internal temperature probing based on FBGs [120]: (**a**) schematic of integrating FBG sensors into the central void of cylindrical jelly-roll Li-ion batteries; (**b**) tracking of solid electrolyte interface (SEI) formation through pressure dynamics data and identification of thermal events (labeled peaks) at C/10 charge/discharge rate, demodulated from Bragg wavelength shifts. (Reproduced with permission from Huang et al. Nature Energy; published by Springer Nature, 2020.).

Parameters other than strain, such as pressure and refractive index changes, have also been explored in lithium-ion batteries for potential health and state estimations with the use of standard and modified FBG sensors. For example, Huang et al. [120] identified the formation of the solid electrolyte interface (SEI) through a combination of a single mode FBG fiber and a micro-structured FBG fiber with enhanced hydraulic pressure sensitivity owing to a high air-filling fraction. Pressure and temperature-induced wavelength signals are discriminated using their respective wavelength shift expressions, similar to the reference sensor methods discussed in Section 4.2. Strain-associated signals were eliminated using the mechanical-decoupling method of placing the fiber sensors in the center void of LFP-based cylindrical cells. The implementation of FBGs is illustrated in Figure 8a. Moreover, as shown in Figure 8b, the peak in temperature and rise in pressure observed as a function of cycling time during the charge of the first cycle agreed well with the formation of SEI, which serves as a strong indicator of the health status of a cell. Health-determining thermodynamic parameters such as enthalpy of formation and entropy can also be calculated using the temperature data combined with isothermal calorimetry and a thermal-equivalent-circuit (TEC) model of the cell. However, extended aging tests with a large number of cycles are still needed to track the growth and decomposition of the SEI, and therefore, estimate the SOH of a cell.

**Figure 9:**
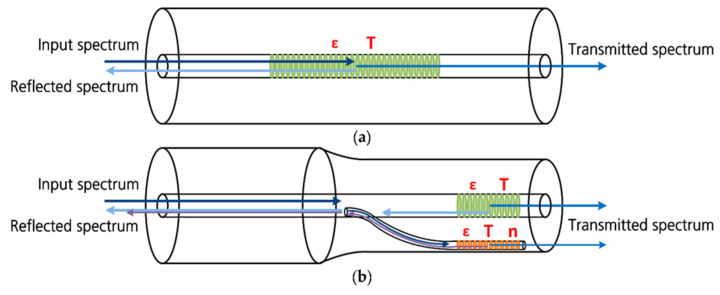
Schematics of typical and sensitivity-enhancing optical waveguide structure [127]: (**a**) the original design where gratings are inscribed into the fiber core; (**b**) the self-compensating design that can enhance refractive index sensitivity by inscribing an additional waveguide in the cladding to divert a portion of the propagating light.

Another work with FBGs measured the refractive index of the electrolyte as a result of the changes in conductive ion concentration [127]. Illustrated in Figure 9, Nedjalkov et al. introduced a novel fiber design: in addition to FBGs in the fiber core, an additional optical waveguide was inscribed within the cladding of the original fiber using the point-by-point method via a femtosecond laser to enhance its sensitivity to refractive index variation in battery ionic solutions. Thus, the fiber core can serve as a temperature or strain-compensating reference, while the additional surface waveguide can serve as a refractive index sensor. This work shed lights on possible methods of using FO sensors to estimate the internal cell resistance based on the electrolyte composition, as resistance represents a crucial factor of SOH estimation other than capacity loss.

**Figure 10:**
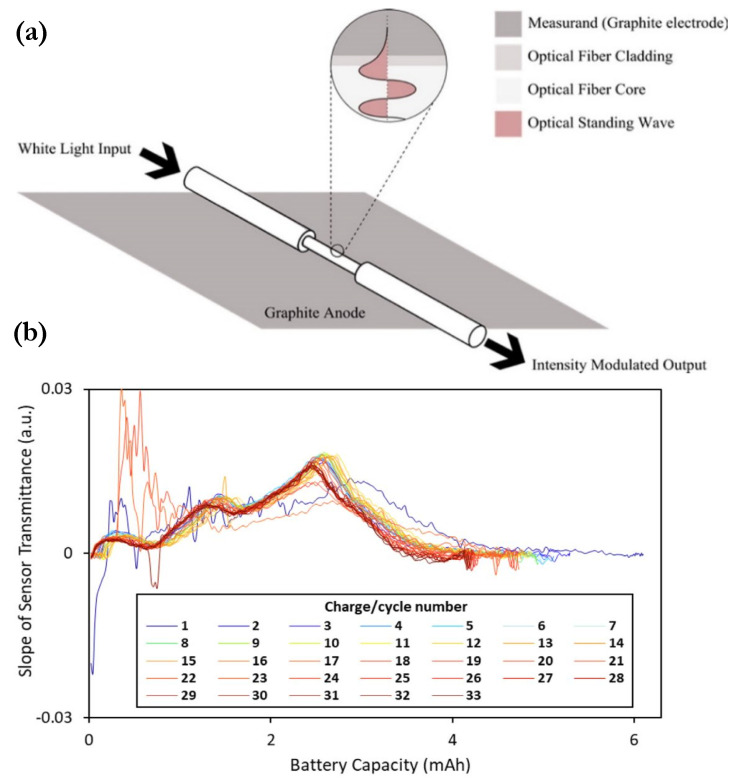
Fiber optic evanescent wave (FOEW) sensor in Li-ion cells: (**a**) schematic of FOEW sensor embedded onto graphite anode and evanescent wave (Reproduced with permission from Ghannoum et al., Applied Materials; published by American Chemical Society, 2016.) [130]; (**b**) correlation between charge capacity and the slope of optical transmittance of the embedded FOEW sensor (Reproduced with permission from Ghannoum et al. Journal of Energy Storage; published by Elsevier, 2020.) [129].

Aside from the extensive research done in the implementation of FBG sensors in batteries, FOEW sensors have also been investigated recently by a few research teams. The possible advantage of FOEW sensors in practical use is their lower overall system cost due to cheaper interrogation at an expense of sacrificing accuracy as compared to FBGs, as discussed previously in Table 1. Ghannoum et al. demonstrated several examples of embedding FOEW-based fibers into the graphite anode of pouch cells [128,129] and Swagelok cells [130,131] to study the correlation between transmittance changes in light spectrum and the SOC or cell capacity. Figure 10a shows a schematic of the FOEW sensor and the exponentially decaying evanescent field in the sensing region. During the sensor fabrication, a layer of glycerol was applied to test the transmission loss at the sensing region to ensure the sensitivity and functionality of etched optical fibers. In their more recent work, Ghannoum et al. explored the possibility of using FOEW sensors for battery SOH monitoring. The SEI formation was captured by the transmittance signals, and the slope of optical transmittance was found to correlate with the battery charge capacity [129]. Figure 10b shows the three major peaks in the transmittance slope spectrum identified as the phase transition stages of the lithiated graphite anode. In addition, the SOC correlation with transmittance signals found by Ghannoum et al. was further explored by Hedman et al. [132]; the LFP cathode was studied using FOEW sensors based on pure silica fiber with the polymer coating removed. Both reflection-based and transmission-based measurements were tested, and the trade-off between optical system set-up complexity and implementation safety was discussed for these two configurations. The intensity changes of the optical fiber were clearly observed in the transmission configuration, as the intensity increased during charging and decreased at discharging of the cell. These works have shown that lower cost FO point sensors, other than FBGs, are possible and likely more economical solutions to enhanced parameter monitoring for BMS.

#### 4.3.3. Acoustic Emission Measurements

Contemporary BMS operate with current-based and OCV-based equivalent-circuit cell models combined with state estimation algorithms such as Kalman filters (KF) and EKF [1,133], in which the estimated values are updated recursively by measurements of cell external parameters. These techniques rely heavily on statistical inference and simulated models that may be far from accurate as the battery ages or operates under extreme conditions, and they also suffer from the inaccuracy of state variable initializations and the sensitivity to process noise that hinders the convergence of the algorithms. Recently, an alternative solution has been demonstrated by using acoustic emission spectroscopy (AES) to directly measure cell internal structural changes through acoustic signal intensities [134]. Not only was acoustic impedance proved to vary as a function of electrode density through acoustic time-of-flight (ToF) analysis, but correlations between acoustic attenuation and elastic modulus and density of electrode layers were also reported. Therefore, cell SOC can be determined by the real-time monitoring of these structural evolutions due to the density changes that occur during the lithiation and de-lithiation of the electrodes. Rather than relying on indirect measurements and estimation-based methods, cell SOC can potentially be measured directly through acoustic emission sensors. Robinson et al. [135] showed the acoustic emission sensing technique with surface measurements on a commercial Li-ion pouch cell. The acoustic intensity variations can be derived as density changes, which directly represent the changes in SOC. The anode and cathode were located by observing the increase and decrease in density in a ToF analysis color map. However, currently, for the studies in electrochemical environments including batteries, these acoustic sensors are usually based on piezoelectric transducers for ultrasound flaw detectors [134,135,136]; fiber optic sensors have yet to be applied. One potential candidate for this purpose is FBG sensors because of the enhanced signal-to-noise ratio that can be achieved [55]. Zhang et al. [137] also demonstrated an acoustic emission sensing system based on a fiber optic FP interferometer that is formed by two cascaded chirped FBGs.

## 5. Parameter Monitoring for Thermal Runaway Detection in Batteries

Thermal runaway in Li-ion batteries is a series of exothermic chain reactions that begins with SEI layer decomposition and concludes with the degradation of the electrode active materials. In the process, the battery temperature can rise to over 500 °C [41]. This section discusses two proposed measurements, spatially distributed temperature and CO_2_ concentration, that can potentially detect this imminent cell failure at an early stage. Preliminary research works have been done to prove the accuracy and effectiveness of deploying optical-fiber-based sensors to address this issue. While the earliest detection of CO_2_ is at 70 °C [138], the onset of thermal runaway, defined as the temperature when the rate of heat generation starts exceeding the rate of heat removal, is typically at 130–150 °C [14,138]. A battery heat release rate of 1 °C/min is typically used as the benchmark. Thermal runaway can be prevented by stopping the charging/discharging of the thermally anomalous cell or by triggering the air/liquid cooling system of the thermal management system within the master controller.

### 5.1. Distributed Temperature Measurements

While discerning cell operational characteristics requires accurate internal temperature measurements by point sensors, an abnormal thermal event in batteries demands distributed temperature measuring methods to pinpoint the temperature hotpot that is otherwise hard to be pre-located by single point measures. In other words, pursuing high temperature accuracy is rather unnecessary when determining the source where thermal runaway occurs is more important. A few studies of battery temperature monitoring have demonstrated the implementation of fully distributed sensors such as Rayleigh or Raman scattering-based FO sensors. Most works have focused on quasi-distributed FBGs. The obvious reason is the high interrogation cost of a fully distributed scheme where the cost-effectiveness ratio can only be justified in applications such as power transmission lines and power plant pipelines where linear characteristics extend for a sensing range up to 10 km and above [10]. Vergori and Yu [108] examined the distributed temperature and strain gradients of two Li-ion pouch cells where 5 m of SMFs based on Rayleigh OFDR were deployed. The temperature gradient profile showed clearly that higher temperatures occurred in the center of the cell while temperatures dropped toward the two edges due to heat dissipation. This result was compared with the deployment of eight thermocouples and concluded that a more complex system setup is required by the electrical sensors as more data acquisition channels and resulting wires are necessary. Another work by Li et al. [139] utilized a Raman OTDR-based system to detect abnormal thermal events in a battery pack. The temperature change can be derived using the intensity ratio of the Stokes and Anti-Stokes scattering lights. However, limited data on temperature-induced signals were shown in this work other than temperature calibration results. 

**Figure 11:**
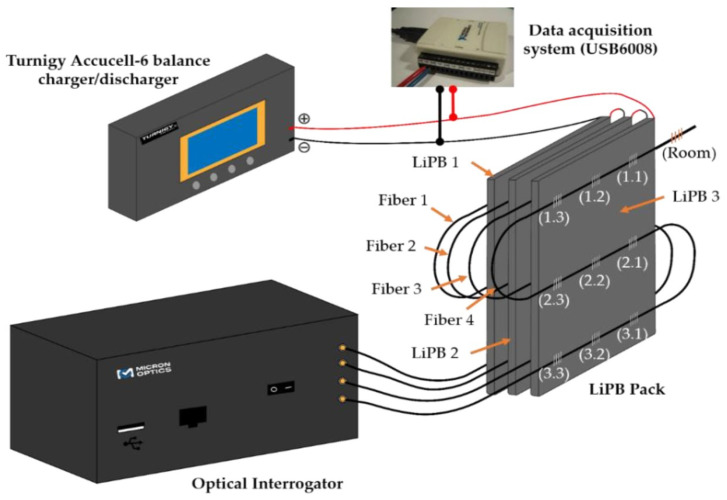
Experimental setup of the quasi-distributed temperature sensing system on a Li-ion battery pack using an FBG network [143].

Several demonstrations of quasi-distributed FBG sensing techniques on batteries have been done. McTurk et al. [140] devised a cell core temperature measurement in a 18650 cylindrical cell with multiple sensing points enabled by FBGs written on a single fiber sealed in fluorinated ethylene propylene. A difference in temperature of 2.5 °C between the core and cathode can was observed. Yang et al. [141] also applied a 7-FBG sensor array on the surface of a series of coin cells to measure the temperature under normal conditions, overcharging, and external short-circuit scenarios. Another work by Nascimento et al. [142] compared a network of three FBGs to three thermocouples on the top and bottom positions of a pouch cell. The FBG sensors were reported to have a 0.05 °C higher accuracy and a rise time 28.2% lower than the thermocouples. Then, the same authors conducted a thermal and spatial mapping of a 3-prismatic-cell battery pack combined in series, as shown in Figure 11, with a total voltage of 11 V and with 37 FBGs on four SMFs [143]. Under constant current charging at 1 C-rate, a hotspot was detected at the interface between two cells that exhibited higher current density than others. The temperature hotspot detection by fiber optic thermal mapping was demonstrated for the first time, which highlights the importance of identifying individual cell anomalies. The work with the largest scale done to date is by Meyer et al. [20], with a 13.8 kWh EV battery pack of 96 NMC-based pouch cells with a maximum voltage of 400 V and current up to 100 A. In this study, 96 FBGs were multiplexed in 14 fibers to simultaneously measure the strain and surface temperature changes during fast charging, abusive overcharging, and accelerated aging tests. In the abuse test scenario, the FBG-attached battery pack was overcharged at temperatures above the normal operating range. An internal short-circuit was forced by the penetration of separators, and a temperature rise to 477 °C was measured during thermal runaway. With proper temperature thresholds established in BMS, thermal runaway could be effectively prevented.

### 5.2. Vent Gas Concentration Measurements

Lithium-ion batteries have a normal operating temperature range of 15 to 45 °C, and the upper temperature limit for storage and operation is typically below 60 °C [144]. If the temperature exceeds a critical level under abusive conditions, thermal runaway can occur and generate a variety of toxic and flammable gases within the cell [145]. When the temperature starts to rise, the initial decomposition of the SEI layer takes place at around 70–90 °C [138]. This composite thin film consists of inorganic species from the decomposition of lithium salts and organic components from the reduction of solvents. The degradation reaction produces CO_2_, O_2_, and some hydrocarbons such as C_2_H_4_. At 100 to 120 °C, the electrolyte begins to decompose and generate CO, CO_2_, H_2_, and more hydrocarbons such as CH_4_, C_2_H_6_, and C_2_H_4_. At 130 to 170 °C, the polymer-based separator reaches its melting point and starts to shrink and melt. Although this is an endothermic reaction, the direct contact of anode and cathode can cause an internal short circuit, generating more heat. The intercalated lithium inside the anode reacts with electrolyte and produces several flammable hydrocarbons such as C_2_H_4_, C_3_H_6_, and C_2_H_6_. At 150 to 180 °C, the active material of the cathode decomposes, generating oxygen that can undergo an exothermic reaction with the electrolyte. The organic electrolyte solvent is oxidized to produce CO, CO_2_, and water vapor. Highly toxic fluorides such as HF and POF_3_ are formed when the decomposition product of electrolyte lithium salt reacts with water vapor. A further reaction of HF with Li+ in the electrolyte produces explosive H_2_ gas. When the temperature increases above 150 to 180 °C, the reaction may become self-sustaining, and the pressure continues to build up within the cell, which brings the potential of rupture or explosion. Based on the mechanism of thermal runaway, it is reasonable and convenient to take the released gas as the characteristic fault signal to monitor and detect the presence of abusive events in lithium-ion batteries [146]. The gas composition and total gas emissions during the thermal runaway process are related to the stage and severity of an abuse event. Therefore, it is crucial to detect the venting gases which not only can provide early warning before failure but also can predict the SOH based on battery degradation models to ensure safe operation of the battery system [147]. 

Conventional analytical instruments such as Fourier Transform Infrared Spectroscopy (FTIR) [148], Gas Chromatography-Mass Spectroscopy (GC-MS) [149], Nuclear Magnetic Resonance (NMR) [150], Ion Chromatography (IC) [12] and Gas Chromatography-Flame Ionization Detector (GC-FID) [151] have been widely applied to analyze the off-gas emission. However, it is difficult to use these techniques to track the gas evolution in real time, and they are extremely expensive. To date, the main approach for the in situ monitoring of thermal runaway focuses on terminal voltage and surface temperature sensing [41]. Gas sensors based on novel materials and transducers have also emerged as in situ monitoring platforms for early fault detection and safety improvement of power assets [152], providing more sensitive and accurate early-stage diagnosis results [153]. Koch et al. employed a set of sensors including semiconducting SnO_2_ sensors that is sensitive to CH_4_, C_3_H_8_, and CO for analysis of the ejected gas during thermal runaway [154]. This gas sensor provided the fastest and clearest signal of thermal runaway among voltage, temperature, pressure, and creep distance sensors. Fiber optic gas sensors offer several advantages over electrical sensors as they can be fabricated to be compatible with electrified environments without the risks of shorts, sparks, or EMI [155]. FBG sensors recording the temperature and the strain distribution on the surface of the cell have been developed for monitoring the aging of the cells and enhanced battery safety. However, the literature addressing the use of fiber optic sensors for the detection of off gassing from Li-ion batteries is still in its infancy. Li et al. designed an in situ monitoring system for an Li-ion battery based on a multifunctional fiber [139], which consisted of distributed temperature, gas, and strain sensing functions. CH_4_, CO, CO_2_, and HF could be detected by tunable diode laser absorption spectroscopy (TDLAS) with an accuracy of 20 ppm according to their wavelength dependency. Lochbaum et al. applied fiber optic colorimetric sensors to gather real-time information on the dynamics of CO_2_ evolution during Li-ion battery overcharge [156]. The gas evolution was irreversible, and the CO_2_ content kept increasing during each overcharge cycle. Both research efforts provide some insights on real-time gas evolution monitoring by fiber optic sensors for the early detection of potentially hazardous cell states.

## 6. Conclusions and Future Perspectives

Li-ion batteries are the leading power source for electric vehicles, hybrid-electric aircraft, and battery-based grid-scale energy storage. These batteries must be actively monitored to enable appropriate control by BMS and early detection of thermal runaway. The most commonly measured parameters in fielded systems include current and voltage using existing electrical connections. Current and voltage measurements enable the calculation of a battery’s SOC and SOH, allowing the BMS to send appropriate charge and discharge commands that will keep it within safe operating limits. In EVs, thermistors are used to monitor battery temperature at the module level. In grid-scale systems, temperature monitoring is typically done at the module rather than at the cell level using thermistors or thermocouples. Emerging technologies include ultrasound [134,135,136] and strain gauges [157,158,159] for general SOC and SOH monitoring and gas detectors [154] for the identification of battery off-gassing prior to thermal runaway.

Fiber optic sensors offer several technical advantages relative to existing battery monitoring technologies. They are immune to EMI and RFI, which readily arise in energy storage systems due to the fast-switching frequency of power converters. Their light weight and flexibility allow embedding in individual cells to monitor internal temperature and physical changes arising from degradation. Such internal measurements are particularly valuable for larger cells and can help reduce the cost of oversizing battery packs, which is a common practice driven by state variable (SOC, SOH, and SOL) estimation uncertainties that exist when measurements are done at the external module level. An insufficient access of energy capacity of 10 to 80% was reported for most commercialized Li-ion battery systems [160]. Furthermore, the high sensitivity, multiplexing capability, and potential for functionalization permit the simultaneous monitoring of a wide range of parameters that indicate battery health. Previous studies have successfully used FO sensors to monitor Li-ion battery temperature, strain, pressure, refractive index, and off-gassing in laboratory settings. This review summarized efforts to distinguish changes caused by different parameters, especially strain and temperature. Assessing the possibilities of integrating FO sensors with next-generation batteries requires careful cell cycle life and abuse testing studies to identify the appropriate failure indicators. For instance, for future high energy density solid-state batteries with Li-metal anodes, FO sensors can potentially be a promising candidate to predict thermal runaway by detecting O_2_ generation from solid electrolytes [161] and to help evaluate the ability of SEI to suppress dendrite formation on the Li-metal anode [162,163] by monitoring the associated strain or pressure changes. 

**Figure 12:**
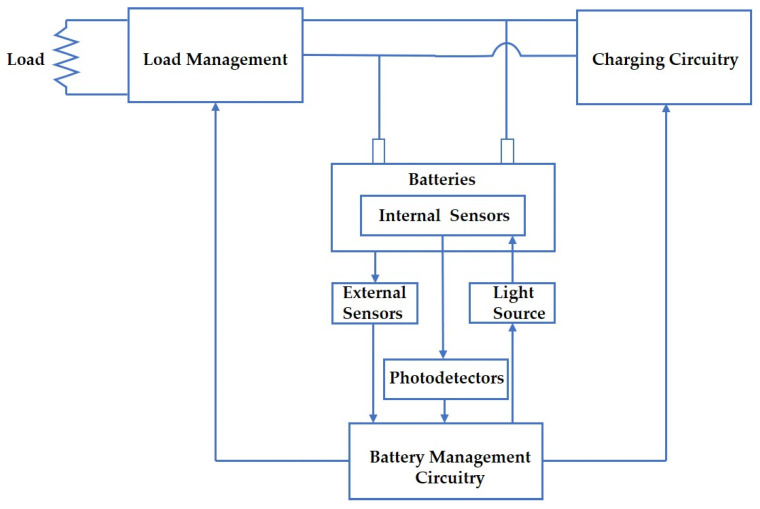
Block diagram of the battery management system with FBG internal sensors and low-cost photodetectors [165].

However, there are still several barriers to the practical implementation of FO sensors in battery energy storage systems. Concerns about cost pose the most substantial roadblock to enhanced monitoring, especially cell-level monitoring. Addressing this concern will require a critical assessment of the value of reduced incidents and predictive maintenance enabled by better data collection during operation [164]. The most expensive components are the light source and interrogator/spectrometer in a fiber optic sensing system. Developments that will further reduce their cost include replacing the traditional costly interrogator with a photodiode detector that can be incorporated into a photonic integrated circuit and using light sources such as light-emitting diodes (LED) that are usually three to six times cheaper than superluminescent diodes (SLD) or the tungsten-based light sources used in laboratory settings. Specifically, Raghavan et al. proposed the design of a wavelength-resolving detector that combines a dispersive-filter coated detector with a position-sensitive photodetector to serve as the demodulation tool [21]. Figure 12 gives the schematic representation of the entire system designed. It was claimed that the FBG-sensing based BMS with this wavelength detector can lower the cost to that of conventional electrical-based sensing systems [21,165]. Another effort in cost reduction by the same group is to inscribe FBGs in MMFs, which typically have a larger core diameter than SMFs. This modification could simplify system configuration and enable the use of less expensive LED light sources because of the exemption from precise alignments [160]. 

A few concerns have also arisen about the insertion safety of optical fibers into batteries and the durability of the materials both on the fiber side and the battery electrode side. Although further efforts will be needed to validate the stability of a fiber-instrumented battery system through long-duration cycling, preliminary experimental results from several groups have shown that a fiber-embedded cell exhibits comparable capacity retention to a pristine cell [21,22,119,120]. Bae et al. also confirmed that the voltage profile of a battery that had an FO sensor implanted within the anode material showed excellent agreement with the one that had the fiber attached on the surface of the anode [126]. A reflection-based optical sensing configuration is preferred for simplified hermetic sealing about the fiber to prevent leakage and exposure to the air [132]. The reflection configuration has both the detector and light source at the same end, allowing for a simpler sealing process with only a single entry for the optical fiber. Lastly, to minimize the risk of damaging fibers during operation, the FO sensors should be characterized and calibrated under the operating temperature and chemical condition of the battery system of interest.

Ultimately, the implementation and utility of FO sensors in batteries will depend on the requirements of the energy storage application. Large-scale energy storage systems could support the higher capital investment for a multiplexed FO interrogation system when the cost is spread across the monitoring of many individual cells and the cost of additional sensing points is low. The complexity of manufacturing and assembly suggest that FO sensors would be better suited for systems with a smaller number of large form factor cells rather than a larger number of small cells. Internal temperature monitoring is also more critical in large form factors given the greater gradient between the external and internal temperature. Furthermore, FO sensors have value for operations that place a premium on safety and early failure detection, such as energy storage systems in electrified aircrafts. A wider range of scales of battery applications can become viable in the future as low-cost FO point sensors, such as FOEW and FP sensors, are commercialized with the integration of low-cost photo-detecting interrogation systems enabled by photonic integrated circuits.

## Figures and Tables

**Table 1:** Quantitative comparison matrix between battery type and scale of application.

Cell Type	Physical Dimensions	Typical Energy Capacity	Cells Required for a 100 MWh Utility-Scale Battery	Cells Required for a 500 kWh Truck Battery Pack	Cells Required for a 65 kWh Passenger EV
Li-ion Cylindrical	18–46 mm diameter, 65–80 mm height [31]	10–17 Wh [30,32]	5,900,000–10,000,000	30,000–50,000	3800–6500
Li-ion Prismatic	Varies—e.g., Samsung SDI94 133 mm × 173 mm × 45 mm [33]	50–350 Wh [30]	286,000–2,000,000	1400–10,000	190–1300
Li-ion Pouch	Varies—e.g., AESC cell 261 mm × 261 mm sheet [34]	200 Wh (typical for EV application) [34,35,36]	500,000	2500	325

**Table 2:** Comparative criteria of commercialized fiber optic sensors relative to other temperature measurement methods for Li-ion batteries.

Sensor Type	Sensitivity ^1^	Accuracy ^1^	Base Cost (System + One Sensor) ^2^	Cost/Sensing Point ^2^	Robustness	Multiple Parameters	Interrogation	Location	Level of Impact for Internal Measurements ^3^
FBG sensor	10–13 pm/°C [43,44]	±0.05 °C [45]	Over $10,000	$165/pt. (EV); $31/pt. (ET); $11/pt. (GES);	Resistant to corrosion, EMI, RFI, high voltages	Temperature, strain, pressure, etc.	Capable of multi-parameter monitoring and signal discrimination	External and internal measurements	Level 2
Other fiber optic point sensor^4^	10 pm/°C [46]	±0.2 °C [47]	≈$4000	-	Resistant to corrosion, EMI, RFI, high voltages	Temperature, strain, chemicals, etc.	Capable of multi-parameter monitoring and signal discrimination	External and internal measurements	Level 2
Thermistor	3–6%/°C [48,49]	±0.01–±0.05 °C [50]	$100~200	-	Prone to electrical noises	No	Nonlinearity between temperature and resistance needs to be compensated	External and internal measurements	Level 3
Resistive temperature detector	0.38%/°C [42]	±0.01–±0.2 °C [50]	≈$200	-	Resistant to corrosion, but prone to electrical noises	No	Linear behavior between temperature and resistance enables simple interrogation	Externally capable but requires thin-film fabrication for internal measures	Level 3
Thermocouple	1–70 µV/°C [51]	±1–±2 °C [51]	≈$400	-	Sensitive to corrosion, low long-term stability	No	Nonlinearity between temperature and voltage needs to be compensated	Externally capable but requires thin-film fabrication for internal measures	Level 3
Electrochemical impedance spectroscopy	Depends on battery chemistries and measurement frequency	±0.17–±2.5 °C [52]	Implementation dependent	-	Strongly interference sensitive	Temperature, other impedance-dependent cell state variables	Interpreting temperature from impedance can be challenging due to the SOC and aging dependency of impedance [42]	External measurements	Level 1
Infrared thermal imaging	≈0.05 °C [50]	±0.1–±5% [50]	Implementation dependent	-	Delicate and non-practical for commercial purposes	No	Interfered by radiations from surrounding objects	External and internal measurements	Level 1
Typical values of sensitivity and accuracy for each category of sensors are used, though actual values depend on specific types in that category. The costs presented are approximated from vendor provided pricing information on off-the-shelf products. Base costs are dominated by the interrogation cost, especially for fiber optic sensors. Calculation is shown in Equation (1) (EV = Electric Vehicle, ET = Electric Truck, GES = Grid-Scale Energy Storage). Potential level of impact of Li-ion batteries is classified into three levels: level 1: non-intrusive with no impact; level 2: minimal impact, capacity retention is comparable to original cell; level 3: negative impact, conventional size of sensors can cause increase in internal impedance and impedes ionic transport, which may result in lithium plating and lead to capacity loss or even internal short-circuit. Fiber optic point sensors other than FBGs includes Fabry–Perot interferometer, fluorescence-based, and evanescent wave field sensors.									

**Table 3:** Proposed applications for different multiplexing schemes of fiber optic (FO) quasi-distributed sensors.

Multiplexing Scheme.	Number of Sensing Points	Scale of Application
Conventional WDM	tens	BMS in passenger EV
WDM/FDM	tens to 100 s	BMS in heavy-duty electric truck
TDM	100 s to 1000 s	Utility-scale energy storage

## Abbreviations

AES	Acoustic Emission Spectroscopy
BMS	Battery Management Systems
CDM	Code-Division Multiplexing
DFOS	Distributed Fiber Optic Sensors
DTS	Distributed Temperature Sensing
EKF	Extended Kalman Filters
EMI	Electromagnetic Interference
ET	Electric Truck
EV	Electric Vehicle
EW	Evanescent Waves
FBG	Fiber Bragg Grating
FDM	Frequency-Division Multiplexing
FO	Fiber Optic
FOEW	Fiber Optic Evanescent Wave
FP	Fabry–Perot
FTIR	Fourier Transform Infrared Spectroscopy
GC-FID	Gas Chromatography-Flame Ionization Detector
GC-MS	Gas Chromatography-Mass Spectroscopy
GES	Grid-Scale Energy Storage
IC	Ion Chromatography
IR	Infrared
KF	Kalman Filters
LFP	Lithium Iron Phosphate
LPG	Long-Period Gratings
MMF	Multi-Mode Fiber
NMR	Nuclear Magnetic Resonance
OCV	Open-Circuit-Voltage
OFDR	Optical Frequency-Domain Reflectometry
OTDR	Optical Time-Domain Reflectometry
RFI	Radio Frequency Interference
RTD	Resistance Temperature Detector
SEI	Solid Electrolyte Interface
SLD	Superluminescent Diodes
SMF	Single Mode Fiber
SNR	Signal-to-Noise Ratio
SOC	State-of-Charge
SOH	State-of-Health
SOL	State-of-Life
TDLAS	Tunable Diode Laser Absorption Spectroscopy
TDM	Time-Division Multiplexing
TEC	Temperature-Equivalent-Circuit
ToF	Time-of-Flight
UV	Ultraviolet
WDM	Wavelength-Division Multiplexing
